# RETRACTION: The Impact of Paravertebral Nerve Blockade on Postoperative Surgical Site Wound Pain Management in Patients Undergoing Video‐Assisted Thoracoscopic Surgery for Pulmonary Carcinoma Resection

**DOI:** 10.1111/iwj.70608

**Published:** 2025-04-21

## Abstract

**RETRACTION:** ZhangL. , ShenJ. , and LuoY. , "The Impact of Paravertebral Nerve Blockade on Postoperative Surgical Site Wound Pain Management in Patients Undergoing Video‐Assisted Thoracoscopic Surgery for Pulmonary Carcinoma Resection,” International Wound Journal 21, no. 4 (2024): e14608, 10.1111/iwj.14608.38151912 PMC10961871

The above article, published online on 27 December 2023, in Wiley Online Library (http://onlinelibrary.wiley.com/), has been retracted by agreement between the journal Editor in Chief, Professor Keith Harding; and John Wiley & Sons Ltd. Following an investigation by the publisher, all parties have concluded that this article was accepted solely on the basis of a compromised peer review process. The editors have therefore decided to retract the article. The authors did not respond to our notice regarding the retraction.

**RETRACTION:** L. Zhang , J. Shen , and Y. Luo , "The Impact of Paravertebral Nerve Blockade on Postoperative Surgical Site Wound Pain Management in Patients Undergoing Video‐Assisted Thoracoscopic Surgery for Pulmonary Carcinoma Resection,” International Wound Journal 21, no. 4 (2024): e14608, 10.1111/iwj.14608.38151912 PMC10961871

The above article, published online on 27 December 2023, in Wiley Online Library (http://onlinelibrary.wiley.com/), has been retracted by agreement between the journal Editor in Chief, Professor Keith Harding; and John Wiley & Sons Ltd. Following an investigation by the publisher, all parties have concluded that this article was accepted solely on the basis of a compromised peer review process. The editors have therefore decided to retract the article. The authors did not respond to our notice regarding the retraction.

